# News from Societies

**Published:** 1954

**Authors:** 


### *News from Societies*

### BRISTOL MEDICO-CHIRURGICAL SOCIETY

The Annual Clinical Meeting of the Society was held at the Bristol Royal Infirmary on 11th November, 1953.

The meeting was well attended and there were nearly forty cases and demonstrations. It is not within the scope of this article to summarize every case, but several interesting and important points were emphasized in the discussion at the end of the meeting.

**Professor Neale** discussed an excellent demonstration of chest deformities in childhood. Now that rickets has virtually been eliminated, it is even more apparent that there are a number of other causes of malformed chests, such as congenital heart disease and congenital anomalies of the thoracic spine and ribs. Professor Neale emphasized the importance of establishing the correct aetiology in these cases.

**Mr. Pridie** demonstrated eight cases of arthrodesis of the hip by central dislocation and made the following points most clearly:

Cases of Osteoarthritis of the hip should not be treated surgically until the patient is unable to work through pain and disability. Arthrodesis of the type demonstrated is the operation of choice. After such a procedure, not only is pain abolished but commonly the patient returns to his occupation which may be most arduous. Arthroplasty with the use of acrylic cups is not a satisfactory procedure and is rapidly falling into disrepute.

**Dr. Apley** commented on some cases of pseudo-hermaphroditism and pointed out that these conditions are much commoner than the majority of us would suppose. He emphasized the importance of early diagnosis of the true sex so that these unfortunate individuals may be brought up in their correct sex. The true sex can always be determined and practitioners should never hesitate to seek specialist advice in these difficult cases and while treatment is often unsatisfactory many social tragedies can be avoided by correct diagnosis.

**Dr. Cosh** spoke on some aspects of diabetic complications with special reference to neuropathy and retinopathy. The latter condition was being seen much more frequently nowadays because many more diabetics live to a ripe old age and **Mr. Garden** pointed out that not only is diabetic retinopathy common, but its course is unaffected by insulin therapy.

The new insulin zinc suspensions were also discussed and will doubtless become a useful addition to the range of insulins. However, in the light of present knowledge they should not be used for haphazard replacement of well tried preparations now in everyday use.

**Mr. Horton** commented on some interesting cases of peripheral vascular disorders treated surgically and it is very gratifying to learn that major reconstruction operations in this field of surgery are proving highly satisfactory.

A most instructive evening ended with acclamation of all those who had gone to considerable trouble to make the meeting such a success.

### SOME REFLECTIONS

BY RIP VAN WINKLE, M.D.

"You know what that is? In a certain place
meet certain doctors to discuss a case
And other matters, such as weather, crops,
potatoes, pumpkins, lager-beer and hops."

OLIVER WENDELL HOLMES

And that, of course, is the trouble with clinical meetings. I don't say I actually spent much time discussing pumpkins or lager-beer, but by the time I'd had a chat with B., whom I haven't seen for a long time, and asked J. about that case I'd sent him, there didn't seem much time left for looking at the patients.

I heard someone say afterwards it is "rather like an agricultural show; you can only hope to see a few of the exhibits". Certainly all the stalls and sideshows seemed to be doing good business. The insulin exhibits attracted great interest and were, I thought, very clear and concise. The Children's Hospital's collection of chest deformities, was the perfect subject for this type of meeting, and was very ably presented.

The refreshments, which gave us a little more time to discuss the equivalents of Oliver Wendell Holmes's weather, potatoes and hops, were most commendably up to standard. The discussions after were . . . well, shall we say, not quite up to the refreshments, but they gave one an opportunity to compare the oratorical styles of one's colleagues.

There was Professor Neale, never still, very erudite, and, to one listener at least, a little incomprehensible; Mr. Pridie, forthright, an enthusiast for his own viewpoint and therefore putting it over well, and finally propounding the great truth, not always remembered, that the only good result is an active working man or woman. Dr. Apley, in conversational style and with the air of one about to be delivered of a slightly *risqué* story: Dr. Cosh, elegant, quiet, economical in words and gestures, and supported by Dr. Ferguson and others in, almost, warning us off the new insulins, these interesting substances being still, as it were, *sub judice*: lastly, Mr. Horton, in physicianly rather than surgeon's style, quietly apologizing that his best case, misdiagnosed and given up as hopeless in the U.S.A., had been discharged cured and could not be shown: and so to the President charmingly enthusing over a "beautiful pair of discs", before closing the meeting with a word of thanks to the organizers which this humble scribe sincerely applauded.

What do the patients at these shows think of it? I've often wondered: now I have one answer at least. As Dr. X left one cubicle, I overheard him say "Extraordinary case, doesn't look a bit like a . . ." I went in and heard the patient whispering to someone with him, "Funny-looking bloke, doesn't look like a doctor at all".

### "MALIGNANT DISEASE: THE PROBLEM OF THE ADVANCED CASE"

**Mr. R. V. Cooke, M.Ch., F.R.C.S.,** spoke to the Bristol Medico-Chirurgical Society at the December meeting on *Malignant Disease: The Problem of the Advanced Case.*

The gradual rise in age of the population is giving larger numbers of sufferers from certain cancers—particularly large bowel—while the breast is still a major problem in women in the prime of life. Here, however, the recent endocrine treatments are an advance—testosterone for the pre-menopausal woman and oestrogens for the elderly. Mr. Cooke also referred to adrenalectomy, which appears to hold hope for the very advanced case with metastases. Oestrogens are of course well known in the treatment of prostatic carcinoma.

The thyroid is a less common problem and would be rarer still were surgery used earlier in goitre swellings. Radioactive iodine held great hopes at first, but it cures only about one case in ten. With endocrine therapy in general, as **Dr. Foss** reminded [illegible] later, expense can be formidable and each case needs very careful thought.

Mr. Cooke paid tribute to radiotherapy, to be used by means of close co-operati[illegible] between all workers.

On the subject of bowel cancer the speaker turned to the purely surgical aspect. He[illegible] only good and radical excision can offer hope. He showed us inspiring examples [illegible] what could be achieved even before the recent advances in anaesthesia and antibio[illegible] therapy.

Even to-day, surgery it seems still has need of the lion hearted.

